# Intelligent Diagnosis Method for Rotating Machinery Using Dictionary Learning and Singular Value Decomposition

**DOI:** 10.3390/s17040689

**Published:** 2017-03-27

**Authors:** Te Han, Dongxiang Jiang, Xiaochen Zhang, Yankui Sun

**Affiliations:** 1 State Key Lab of Control and Simulation of Power Systems and Generation Equipment, Department of Thermal Engineering, Tsinghua University, Beijing 100084, China; hant15@mails.tsinghua.edu.cn (T.H.); zhangxch2008@mail.tsinghua.edu.cn (X.Z.); 2 Department of Computer Science and Technology, Tsinghua University, Beijing 100084, China; syk@mail.tsinghua.edu.cn

**Keywords:** rotating machinery, condition monitoring, intelligent diagnosis, dictionary learning, singular value decomposition, dimensionality reduction

## Abstract

Rotating machinery is widely used in industrial applications. With the trend towards more precise and more critical operating conditions, mechanical failures may easily occur. Condition monitoring and fault diagnosis (CMFD) technology is an effective tool to enhance the reliability and security of rotating machinery. In this paper, an intelligent fault diagnosis method based on dictionary learning and singular value decomposition (SVD) is proposed. First, the dictionary learning scheme is capable of generating an adaptive dictionary whose atoms reveal the underlying structure of raw signals. Essentially, dictionary learning is employed as an adaptive feature extraction method regardless of any prior knowledge. Second, the singular value sequence of learned dictionary matrix is served to extract feature vector. Generally, since the vector is of high dimensionality, a simple and practical principal component analysis (PCA) is applied to reduce dimensionality. Finally, the *K*-nearest neighbor (KNN) algorithm is adopted for identification and classification of fault patterns automatically. Two experimental case studies are investigated to corroborate the effectiveness of the proposed method in intelligent diagnosis of rotating machinery faults. The comparison analysis validates that the dictionary learning-based matrix construction approach outperforms the mode decomposition-based methods in terms of capacity and adaptability for feature extraction.

## 1. Introduction

As a type of equipment widely used in modern industry, rotating machinery is becoming more precise and with more complicated structures. The operating conditions have also become more severe, involving high speeds, high loads, high temperatures, etc. Mechanical sub-systems in rotating machinery, especially the critical components such as bearings [1], gearbox [2], rotor [3] and fan [4] are easily subject to failure, resulting in unexpected downtime losses or even disastrous accidents. Condition monitoring and fault diagnosis (CMFD) technology is a promising tool to realize early fault alarms and minimize losses.

Among the various approaches used in CMFD technology, the signal-based diagnosis approaches and data-driven diagnosis approaches attract continuous interest [5,6]. In signal-based approaches, the foundation is that the fault information can be reflected in the monitored signals, and a diagnosis result can be made by checking the consistency between real-time data and healthy signal patterns. Numerous studies have developed diagnosis algorithms using advanced signal processing techniques in attempts to enhance diagnostic performance. Intense interest is focused on time-domain analysis, frequency-domain analysis, time-frequency analysis [7], wavelet transform [8], empirical mode decomposition (EMD) [9], spectral kurtosis [10] and other techniques. Despite the achieved success, the analysis procedure always needs to inspect the time waveforms and frequency spectra. Hence, the diagnosis decisions largely rely on the expertise of operators and the prior knowledge of healthy signal patterns, which increases the difficulty to popularize this approach for practical industrial tasks [11]. Owing to a large volume of handily available performance history databases, data-driven approaches have been widely exploited in engineering applications. With the aid of artificial intelligence techniques, data-driven approaches extract the underlying knowledge of complicated industrial systems from their historical data. The classification model can be trained by the feature set extracted from data, so as to identify the fault patterns intelligently when similar faults occur afterwards. From this point of view, this diagnosis strategy is also referred as intelligent diagnosis [12]. In general, whether the extracted features are sensitive to mechanical faults plays a key role in the diagnosis performance. According to existing studies, most reported features mainly include the statistical characteristics, the indices related to entropy, the energy ratios, etc. [13,14,15,16]. Due to the lack of comprehensive exploration of signal characteristic, it is often difficult to ensure these features can properly reflect the patterns of raw signal, and the diagnosis ability may be thus degraded. There is, in particular, a need to attempt more efficient feature extraction methods to facilitate the intelligent diagnosis of mechanical failures, especially those can adaptively learn the inherent structures and scales of signal.

With their successful application in image processing [17,18,19], sparse representation (SR) theory and dictionary learning have drawn attention in the field of machinery fault diagnosis in recent years [20,21,22,23,24]. The fundamental idea of SR theory is that natural signals can be represented by a sparse linear combination of atoms in a fixed dictionary [25,26]. As the manually predefined dictionaries may not well match the characteristics of decomposed signals, the sparsity level of representation coefficients will be unsatisfactory and the redundant information will be kept. Many efforts have been devoted to developing adaptive dictionary learning algorithms from raw signals [27,28]. Essentially, dictionary learning provides self-adaptive tools for extracting and analyzing the characteristics of machinery signals. Chen et al. proposed a noise reduction method using adaptive dictionary learning to extract impulse characteristics for bearing and gearbox diagnosis [20]. Tang et al. applied shift-invariant dictionary learning to detect the latent components and separate the fault-related time series from original signals [21]. Most of the previous works preliminarily explored the dictionary learning in signal decomposition, noise reduction and fault signature enhancement, while further studies of its application to intelligent diagnosis are needed. Liu et al. employed the representation coefficients on the learned dictionary as the adaptive features, and achieved high classification performance with a linear discriminant analysis (LDA) classifier for the bearing experiments [22]. Zhou et al. presented a new approach by means of dictionary learning, energy ratios of latent components and hidden Markov model (HMM) for diagnosing bearing faults [23].

As a powerful signal processing technique, singular value decomposition (SVD) has exhibited excellent performance in mechanical fault diagnosis [29,30,31,32,33,34,35]. Different from the conventional feature organization algorithms, the extraction of singular values helps decompose a feature matrix, which guarantees the stability of the features on the basis of matrix theory. The small fluctuations of the matrix elements cause almost no disturbances on the singular value. Also, the singular value possesses another two favorable properties, namely the scale invariance and rotation invariance. Furthermore, as reported in [30,31], the variation trend of a singular value sequence is associated with the energy distribution and complexity of components signals in Hankel-based matrix. In general, the singular value denotes the natural characteristic of matrix. Consequently, this feature is sensitive and qualified to assess the conditions of machinery signals. However, the selection of matrix construction parameters, i.e., lag time and embedding dimension, is tough to date [32]. To tackle this problem, many scholars have applied the adaptive time series decomposition (ATSD) algorithms, such as EMD, local characteristic-scale decomposition (LCD), local mean decomposition (LMD), etc., to decompose the signals and get a finite number of components, which contain the different frequency information from high to low [32,33,34]. Then, the initial feature matrix can be formed automatically by merging these components. Actually, the singular values of the matrix based on this strategy mainly reflect the division states of frequency bands by ATSD. When the signals in different conditions have similar spectral contents, this feature extraction strategy may not distinguish the condition patterns exactly [35].

In this paper, a novel framework using dictionary learning and SVD is proposed for intelligent diagnosis in rotating machinery. We investigate the potential of introducing the dictionary learning scheme as the initial feature matrix extraction method to achieve improved sensitivity and diagnosis capability of singular values. Specifically, the learned dictionary can reveal the abundantly inherent information of the analyzed signal, leading to an expected feature space. By applying SVD to the dictionary matrices, the singular value sequences can be obtained and serve as the feature vectors of raw signals. Due to the high dimensionality of feature vectors, principal component analysis (PCA) is chosen to reduce the dimensionality and improve the discriminability [36,37]. The fault patterns can be visually observed from the scatter plots of the first two or three principal components, and the intelligent diagnosis results can be made by the *K*-nearest neighbor (KNN) algorithm. In addition, the effectiveness and superiority of the proposed feature extraction strategy is investigated in comparison with that of the traditional EMD-based method.

The remainder of this work is organized as follows: the dictionary learning scheme and SVD are reviewed in Section 2 and Section 3, respectively. Then, an intelligent monitoring and diagnosis method of rotating machinery using dictionary learning and SVD is proposed in Section 4. Section 5 contains the description of two fault datasets from bearing and gearbox, the diagnosis procedures, the discussion and comparison of the results. Finally, the conclusions can be drawn in Section 6.

## 2. Dictionary Learning Scheme

### 2.1. Sparse Representation Theory

The basic idea of sparse representation theory assumes that a digital signal can be represented by a sparse linear combination of the atoms, which are from a fixed over-complete dictionary. Generally speaking, for an input signal $y\in R^{n}$, it can be expressed as:

(1) y=Dx+ξ

where $D\in R^{n\times K}$ is a matrix called dictionary, which contains K atoms $d_{i}\in R^{n},\;i=1,\;\ldots \;,K$ as its columns, $x\in R^{K}$ is the sparse representation coefficient, and ξ is assumed as additive noise. When the dictionary D and input signal y are fixed, we hope to obtain the succinct representation coefficient, which means the majority of the entries in coefficient vector are zero or close to zero. That is, only a small proportion of atoms will contribute to approximating the input signal. To measure sparsity level of coefficient vector x, the $l^{0}$-norms of vector *x* can be calculated as follow:

(2) $${\left||x|\right|}_{0}=\sum_{j=1}^{k}{\left|x_{j}\right|}^{0}$$

which represents the number of nonzero items in x. Then the sparest representation can be transformed to the following optimization problem:

(3) $$(P_{0,\;\epsilon })\;\underset{x}{min}{\left||x|\right|}_{0}\;subject\;to\;{\left||y-Dx|\right|}_{2}\leq \epsilon$$

where the approximation error is assessed by $l^{2}$-norm and ϵ is the parameter which depends on the noisy level of signal. The optimization process is generally called sparse coding. For the overcomplete dictionary, Equation (3) are underdetermined systems of equation. This is a combinational optimization problem and the process of sparse coding is a typical non-deterministic polynomial (NP) hard problem. Thus, scholars turn to approximate algorithms to look for the sparsest collection.

The matching pursuit (MP) and orthogonal matching pursuit (OMP) are two simplest but efficient greedy methods. These approaches select the atoms in sequence, according to the correlation between the columns of dictionary and residual signal. Since sparse coding is an indispensable step in dictionary learning scheme, the primary ideas of OMP is introduced briefly. Firstly, the algorithm defines an initial residual ${\hat{r}}^{\left(0\right)}=y$ and a current linear combination of the atoms ${\hat{y}}^{\left(0\right)}=0$; Then, for k=1,… , the ${\hat{r}}^{\left(k\right)}$ and ${\hat{y}}^{\left(k\right)}$ are updated step by step, always keeping $y={\hat{r}}^{\left(k\right)}+{\hat{y}}^{\left(k\right)}$; All the atoms in dictionary D are normalized, that is, ${\left||d_{i}|\right|}_{2}=1$. During kth update, an atom which has a maximum correlation with ${\hat{r}}^{\left(k-1\right)}$ is selected and added to current linear combination. The correlation between atoms and residual can be measured by the following equation:

(4) $$i_{k}=argmax_{1\leq i\leq K}\left|\langle r^{k-1},d_{i}\rangle \right|$$

In this stage, a current linear combination of the atoms ${\hat{y}}^{\left(k\right)}$ can be obtained:

(5) $${\hat{y}}^{\left(k\right)}=\sum_{l=1}^{k}{a_{i}^{k}}_{l}{d_{i}}_{l}$$

where the coefficients $a_{i}^{k}$ can be determined by using least squares methods, that is, minimize ${\left||y-{\hat{y}}^{\left(k\right)}|\right|}^{2}$. Also, an updated residual ${\hat{r}}^{\left(k\right)}=y-{\hat{y}}^{\left(k\right)}$ should be used in the next iteration. This iteration can be repeated until the residual satisfies some set threshold or the number of nonzero elements in x has reach an upper limit. The OMP could generate highly sparse solutions in sparse coding stage and is further adopted in this paper.

### 2.2. K-SVD Dictionary Learning

Sparse representation differs from other conventional basis representation models because the dictionary can provide a wider array of basis functions. This benefit offers more flexibility in signal representation, and thus more validity dealing with tasks like signal compression, feature extraction and more. It should be noted that two challenges exist in this model, one involving the sparse coefficients solving as aforementioned, and the other, designing the dictionary to fit the structure in the analyzed data. The early dictionaries are chosen as a prespecified set of basis functions, such as discrete cosine transforms (DCT), wavelets, curvelets, short-time Fourier transforms, etc. This dictionary designing scheme is simple and always lead to fast algorithms, while the performance largely depends on how adaptive the atoms are to sparsely represent the input signals, indicating it is necessary to manual find the signal pattern firstly. Another route for designing dictionary is to adapt the dictionary with respect to a set of sample signals based on learning. Such dictionary learning process can capture the inherent characteristic of raw signal as the learned atoms, which is essentially an adaptive methods regardless of any prior knowledge of signals.

K-singular value decomposition (K-SVD) is a highly efficient dictionary learning method. This algorithm mainly include two phases. One step is to find a sparse coefficient given a fixed dictionary which can be regarded as sparse coding. Another step is dictionary update stage based on the acquired coefficient vectors. The columns of dictionary are updated sequentially in K-SVD. The pivotal steps of this method will be illustrated briefly.

Given a signal set $Y={\left\{y_{i}\right\}}_{i=1}^{N},\;y_{i}\in R^{n}$ and an initial dictionary $D\in R^{n\times K}$, the sparse representation coefficient vectors $x_{i}$ corresponding to signal samples $y_{i}$ can be gathered to construct the coefficient matrix $X\in R^{K\times N}$. In this problem, we want to get the sparse representation of sample signals Y based on the changing dictionary D and coefficient matrix X. A desirable dictionary, which can decompose the signals sparsely, should be learned in this procedure. The problem can be met by considering:

(6) $$\underset{D,X}{min}\overset{N}{\underset{i=1}{\sum }}{\left||x_{i}|\right|}_{0}\;subject\;to\;{\left||Y-DX|\right|}_{F}^{2}\leq \epsilon ,$$

where ϵ is the reconstruction error and the Frobenius norm of a matrix ${\left||A|\right|}_{F}$ is defined as ${\left||A|\right|}_{F}={\left(\sum_{ij}A_{ij}^{2}\right)}^{1/2}$. Firstly, the D is fixed and an optimal coefficient matrix X can be determined. Then, a second step is conducted to update the dictionary. Here, only one column $d_{k}$ is modified at one time, while other columns in D are freezed. Also, the corresponding coefficient vector $x_{T}^{k}$ which is the kth row in X is changed. We just need to minimize the target function ${\left||Y-DX|\right|}_{F}^{2}$ to realize the atom updating. This process can be expressed as:

(7) $${\left||Y-DX|\right|}_{F}^{2}={\left||Y-\overset{K}{\underset{j=1}{\sum }}d_{j}x_{T}^{j}|\right|}_{F}^{2}={\left||(Y-\underset{j\neq k}{\sum }d_{j}x_{T}^{j})-d_{k}x_{T}^{k}|\right|}_{F}^{2}={\left||E_{k}-d_{k}x_{T}^{k}|\right|}_{F}^{2},$$

where $E_{k}$ means the error for all signal samples when the atom $d_{k}$ is removed. Now, we may intend to perform singular value decomposition (SVD) on $E_{k}$ and find alternative $d_{k}$ and $x_{T}^{k}$ to reduce error. However, it is to be noted that, a step must be done before SVD to make sure the updated $x_{T}^{k}$ is not filled. A variable is define as:

(8) $$\omega_{k}=\{i|1\leq i\leq K,\;x_{T}^{k}\left(i\right)\neq 0\}$$

The sample signals $\left\{y_{i}\right\}$ that use the atom $d_{k}$ can be indexed by $\omega_{k}$ and the positions of nonzero entries in $x_{T}^{k}$ can be determined by parameter i in Equation (9). Define $\Omega_{k}$ as a matrix of size $N\times \left|\omega_{k}\right|$, with ones on the $\left(\omega_{k}\left(i\right),i\right)\mathrm{th}$ entries and zeros elsewhere. The multiplication $x_{R}^{k}=x_{T}^{k}\Omega_{k}$ changes the length of $x_{T}^{k}$ to $\left|\omega_{k}\right|$ by removing the zero entries. Similarly, the matrixes $Y_{k}^{R}=Y\Omega_{k},\;Y_{k}^{R}\in R^{n\times \left|\omega_{k}\right|}$ and $E_{k}^{R}=E_{k}\Omega_{k},\;E_{k}^{R}\in R^{n\times \left|\omega_{k}\right|}$ only include the sample signals or error columns that use the atom $d_{k}$. Therefore, we define:

(9) $${\left||E_{k}\Omega_{k}-d_{k}x_{T}^{k}\Omega_{k}|\right|}_{F}^{2}={\left||E_{k}^{R}-d_{k}x_{R}^{k}|\right|}_{F}^{2}$$

**Figure 1:**
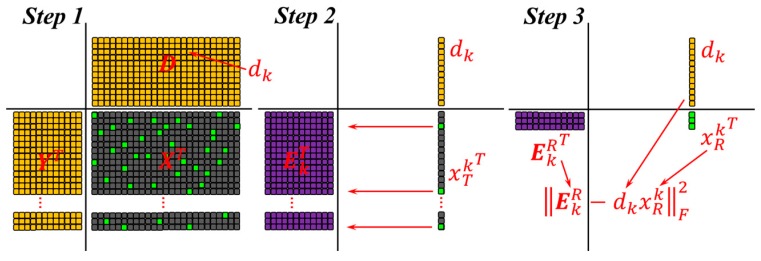
Key steps in K-SVD dictionary learning.

Figure 1 presents a more understandable explanation of this procedure. This time, we can process $E_{k}^{R}=U\Delta V^{T}$ via SVD. After that, the $d_{k}$ can be changed to the first column of U and $x_{R}^{k}$ can be replaced by the first column of V multiplied by Δ(1,1). All the atoms in D are updated one by one and the iteration of sparse coding and dictionary learning is repeated until convergence or the number of iteration reached. Detailed algorithm of K-SVD is given in [27].

## 3. Singular Value Decomposition

According to matrix theory, a matrix A ($A\in R^{m\times n}$) can be decomposed into a number of elementary matrices satisfying mutually orthogonal and unit-rank by SVD, that is:

(10) $$\{\begin{array}{c} A=U\left[\begin{array}{cc} \Sigma & 0\\ 0 & 0 \end{array}\right]V^{T}\;\\ \Sigma =diag\left(\lambda_{1},\lambda_{2},\ldots ,\lambda_{r}\right),\;\lambda_{1}\geq \lambda_{2}\geq \ldots \geq \lambda_{r}\geq 0 \end{array}$$

where $U\in R^{m\times m}$ and $V\in R^{n\times n}$ are two orthogonal matrices, Σ is a diagonal matrix, $\lambda_{i}\;\left(i=1,2,\ldots ,r\right)$ is the singular value of matrix A. Both the value and variation trend of singular value sequence reflect the nature characteristic of matrix. Consequently, the singular value can be used to describe the important information implied in the matrix. Prior to performing SVD, the feature matrix are traditionally formed based on phase space reconstruction technique. Two reconstruction parameters, namely the lag time and embedding dimension, have an influence on the initial feature matrix and further the results of singular value. Unfortunately, no mature theory can be applied to guide the selection of reconstruction parameters. As a result, the further studies focus on the extraction of feature matrix which can represent the nonlinear and non-stationary characteristics of raw vibration signals.

## 4. Proposed Method

In essence, dictionary learning allows more flexibility to extract and analyze the inherent characteristics of signal, regardless of any prior knowledge. The learning process adapts the basic atoms in a dictionary of the characteristic patterns of the monitored signals. The underlying structures and scales of signal, such as periodic impulses and resonance information, can be captured by the basis atoms. Thus the learned dictionary contains a substantial amount of signature information, delivering potential benefits in the feature extraction. For this purpose, we propose to introduce the dictionary learning scheme as the initial feature matrix extraction method for further analysis. The detailed steps of dictionary learning are described as follows:

(1). Given a signal sample, partition the signal into an amount of overlapping segments and generate the dataset Y for dictionary learning.
(2). Set the initial parameters, i.e., initial dictionary $D_{0}$, iteration number K, noise level ϵ.
(3). Sparse coding: use the OMP algorithm in Section 2.1 to find the sparse representation coefficient.
(4). Dictionary update: update the atoms in the dictionary according to the learning algorithm in Section 2.2.
(5). Repeat the step (3) and (4) until the number of iteration reach.

**Figure 2:**
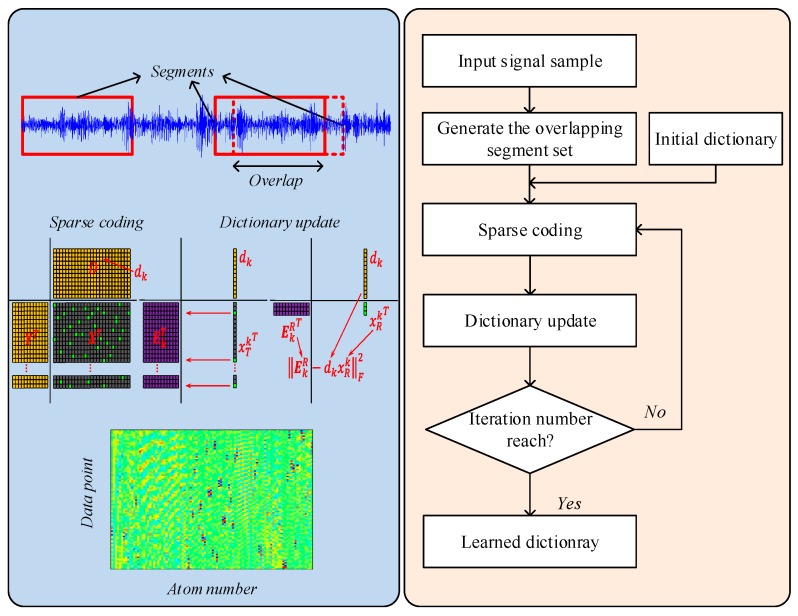
Workflow of dictionary learning algorithm.

A more intuitional workflow is illustrated in Figure 2. The tuned parameters in K-SVD dictionary learning are given in Table 1. With the above steps, the characteristic patterns in the signal sample can be fully explored and mined. The dictionary matrix which contains abundant diagnostic information can be adaptively learned.

**Figure 3:**
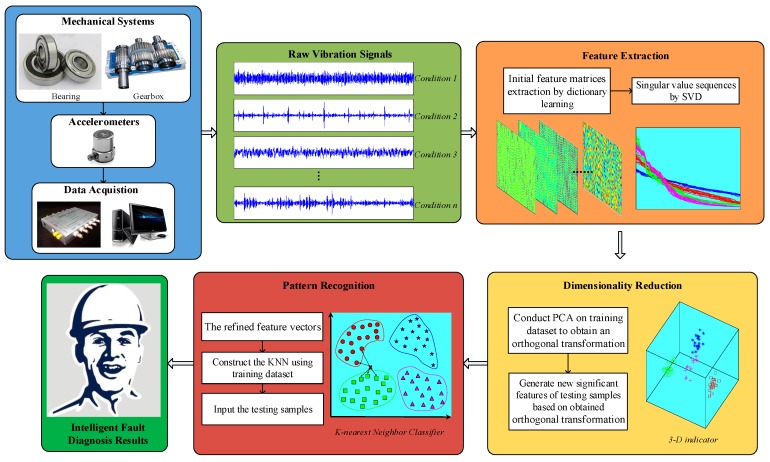
Block diagram of the overview of the proposed approach.

Following the data-driven diagnostic procedure, a novel intelligent diagnosis method for rotating machinery is proposed in this work (see Figure 3). At the first step, the sensitive features should be extracted based on acquired vibration signals to represent different machinery conditions. This step relies on two stages: dictionary learning and singular value extraction. Dictionary learning is employed to extract initial feature matrix. Then, the singular value sequence of learned dictionary matrix can be used as the feature vector of analyzed signal. Technically, the singular value sequence is of high-dimensionality, implying it is impossible to directly serve as the input for classification model. Hence, the next step is dimensionality reduction, which mainly includes linear and nonlinear methods. Although nonlinear dimensionality reduction methods achieved some successful cases in fault diagnosis, it should be noted that the nonlinear methods suffer from some disadvantages such as the computational burden and estimation errors [37]. Otherwise, the nonlinear methods may not outperform the traditional linear ones according to some numerical experiments as reported in literatures [38]. This framework is not limited to a specific dimensionality reduction approach, as linear and nonlinear methods may work in different scenarios. For simplicity, this work just apply a basic approach, PCA, to reduce dimensionality and map singular value sequence to low-dimensional principal components for pattern recognition. Finally, the *K*-nearest neighbor (KNN) classifier is utilized to identify the different machinery conditions automatically. Unlike artificial neural networks (ANN) and support vector machine (SVM), KNN is a non-parametric classification model and the training process is to store the training samples directly, which avoids the cost of parameters tuning and model training in other algorithms.

## 5. Experimental Results and Comparisons

To demonstrate the applicability and superiority of proposed method, the analysis of two fault datasets from bearings and gearbox are conducted, respectively.

### 5.1. Case 1: Rolling Bearing Fault Diagnosis

#### 5.1.1. Experimental Data

The rolling bearing dataset is from the Electrical Engineering lab at Case Western Reserve University. Many scholars have utilized this dataset as a standard reference to test their algorithms over the last decade. The experiment platform consists of a 2 hp motor, a torque transducer, a dynamometer, and control electronics. The test bearings in SKF6205 type support the motor shaft (Svenska Kullagerfabriken AB, Gothenburgh, Sweden). A normal condition and three fault conditions, i.e., inner race fault, ball fault and outer race fault, are tested, during which the vibration signal of bearing can be collected by acceleration sensors with a sampling frequency of 12 kHz. The single point faults are introduced to the test bearings with a fault diameter of 7 mils. The experimental speed is 1772 rpm.

**Figure 4:**
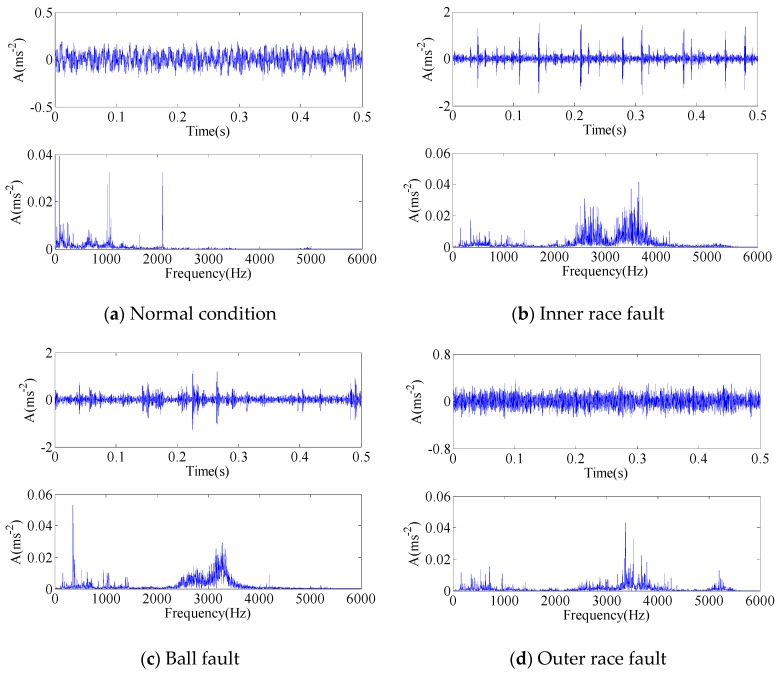
The time waveforms and frequency spectra of vibration signals: (**a**) Normal condition; (**b**) Inner race fault; (**c**) Ball fault; (**d**) Outer race fault.

In each condition, a large sample are divided into 50 samples, each of which contains 6000 data points. 20 samples selected randomly are used for training dataset and the remaining 30 samples are used to test the recognition rate. Totally, there are 80 samples for training and 120 samples for testing in the four bearing conditions. The time waveforms and frequency spectra of the signal samples in the four conditions are presented in Figure 4.

#### 5.1.2. Diagnosis Procedure and Results

As presented previously, the dictionary learning scheme is firstly adopted to generate the initial feature matrices in this work. Each dictionary with a size of 100×100 is learned from each sample, which means 100 atoms, each with the length of 100 points. In principle, increasing the atom length maps directly to the computational burden and reduces the learning capacity based on existing dictionary learning algorithms. For fault recognition of rotating machinery, setting atom length large enough to include one impact will allow more evident fault patterns to be contained by learned dictionary. In our experiments, it can be find each impact lasts less than 100 points in most cases, if any.

**Figure 5:**
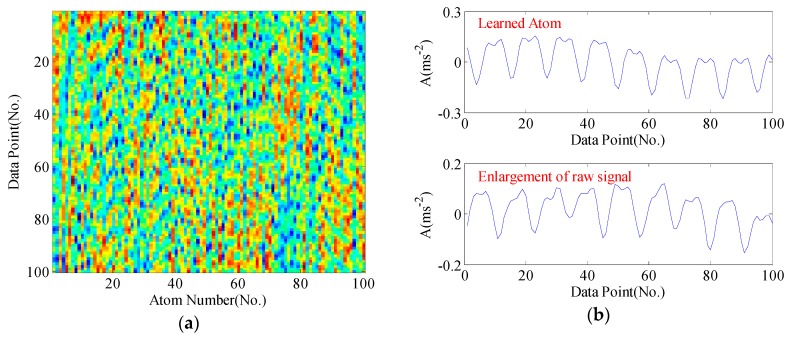
The learned dictionary from the sample signal of normal condition: (**a**) the learned dictionary matrix; (**b**) the learned atom and enlargement of the raw signal.

**Figure 6:**
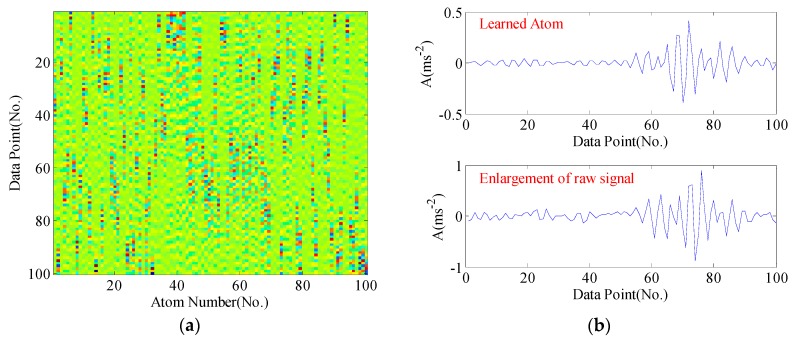
The learned dictionary from the sample signal of inner race fault: (**a**) the learned dictionary matrix; (**b**) the learned atom and enlargement of the raw signal.

**Figure 7:**
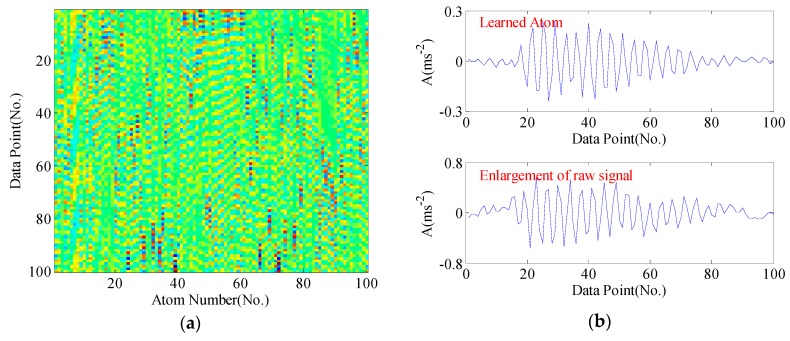
The learned dictionary from the sample signal of ball fault: (**a**) the learned dictionary matrix; (**b**) the learned atom and enlargement of the raw signal.

**Figure 8:**
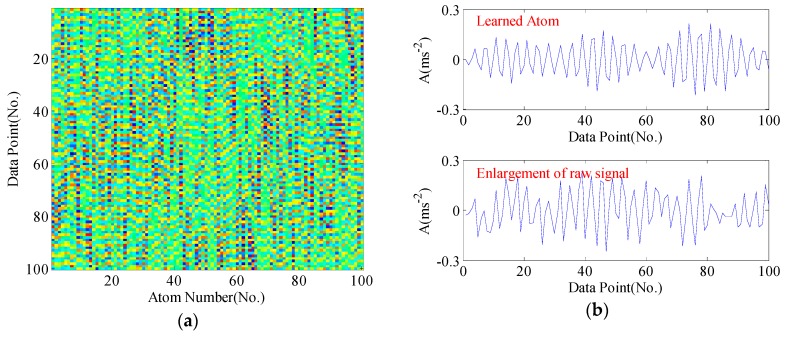
The learned dictionary from the sample signal of outer race fault: (**a**) the learned dictionary matrix; (**b**) the learned atom and enlargement of the raw signal.

For clarity, the dictionary matrices corresponding to four bearing conditions are shown from Figure 5, Figure 6, Figure 7 and Figure 8, respectively. We compare some selected atoms in the dictionary with the raw signal enlargements. One can perspicuously observe the atoms have caught the underlying structure of raw signal. In normal condition, no obvious impacts appear in the learned atoms, whereas acute impulses phenomenon can be noticed in fault conditions. Generally, the mechanisms of generating impulses may have differences when faults occur in different position. Hence, these impulses exhibit different fault signatures, which can be utilized for pattern recognition.

**Figure 9:**
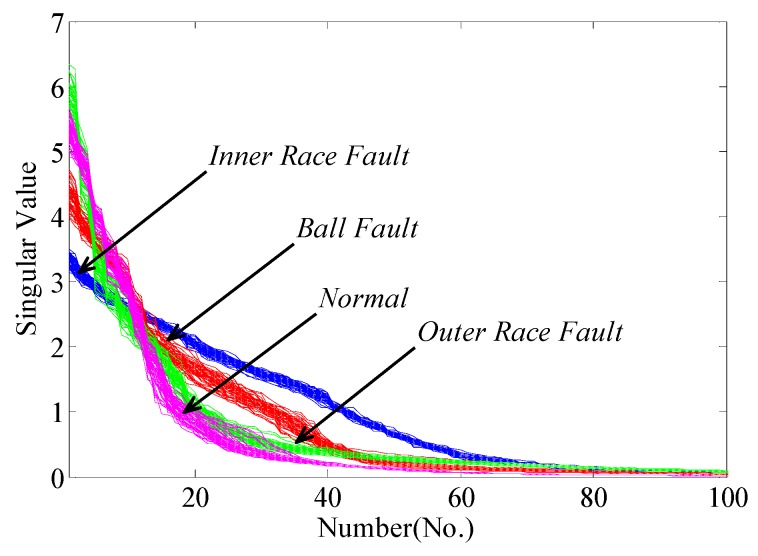
Singular value sequences corresponding to four bearing conditions.

**Figure 10:**
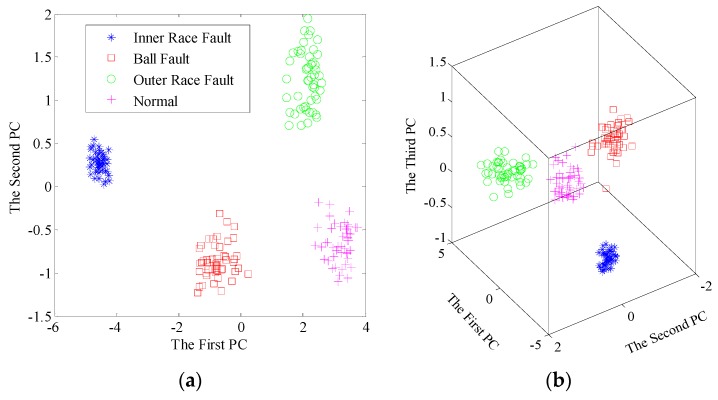
Scatter plots of the principal components after dimensionality reduction using dictionary learning and SVD: (**a**) the first two principal components; (**b**) the first three principal components.

**Figure 11:**
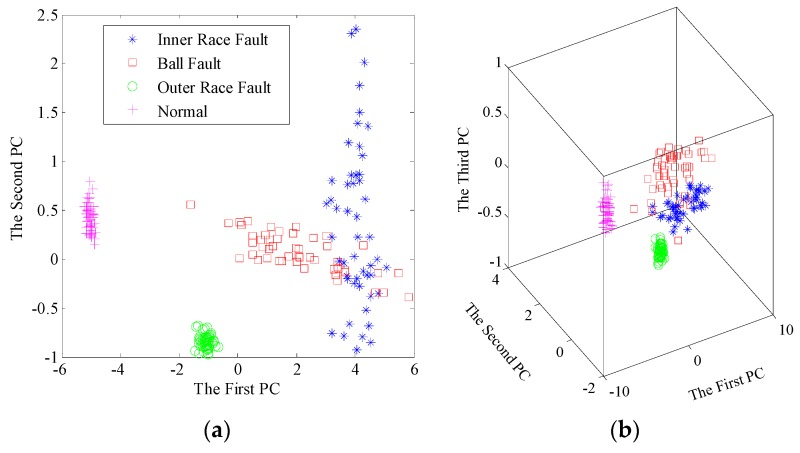
Scatter plots of the principal components after dimensionality reduction using EMD and SVD: (**a**) The first two principal components; (**b**) The first three principal components.

Then, the singular value sequences for total 200 samples are extracted from the learned dictionary matrices. As shown in Figure 9, it is easy to find the gaps of singular value sequences between different bearing conditions. The variation trends of sequences have excellent separability so that fault diagnosis using singular value sequences is positively tenable. Furthermore, the new significant features by PCA, namely the principal components, are adopted to recognize and classify the bearing conditions. To compare the proposed method with traditional EMD-based method, we apply EMD to decompose the sample signals, construct feature matrices and extract singular value sequences. Here, we present the results after dimensionality reduction. The contribution rate of first few principal components are listed in Table 2. The scatter plots of the principal components using the two pre-processors are shown in Figure 10 and Figure 11, respectively.

In both methods, the first two principal components account for up to 95% of the contribution rate, indicating the reliability of first two or three principal components. From Figure 10, all the samples are well separated in 2D and 3D space based on dictionary learning and SVD. Nevertheless, there is an overlapping area between the inner race fault and ball fault, using EMD-SVD pre-processing from Figure 11.

Through inspecting the time waveforms and frequency spectra in Figure 4, we can find the frequency components are relatively similar for the two conditions, leading to the similarity of the frequency band partition. Thus, the major elements in IMF matrices tend to be the same in the two conditions, which results in the overlapping area of principal components. The feature matrix based on this strategy cannot directly reflect the detailed information of vibration signal.

**Figure 12:**
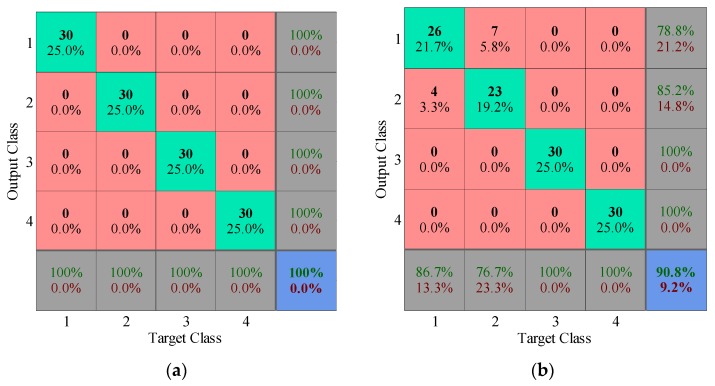
Multi-class confusion matrices of testing samples: (**a**) Dictionary learning pre-processor; (**b**) EMD pre-processor.

The first three principal components are selected as the feature vector. The 80 training samples are applied to construct KNN classifier. The diagnosis results of 120 testing samples are given in Figure 12. The multi-class confusion matrix illustrates the detailed recognition accuracy and misclassified error for all conditions (inner race fault, ball fault, outer race fault and normal correspond to labels 1, 2, 3 and 4, respectively). Undoubtedly, the diagnosis accuracy using dictionary learning is 100%, while EMD pre-processor only achieves the 90.8% accuracy, which the particular misclassified errors keep consistent with the analysis of principal components in 2D and 3D space.

### 5.2. Case 2: Gearbox Fault Diagnosis Results

#### 5.2.1. Experimental Data

**Figure 13:**
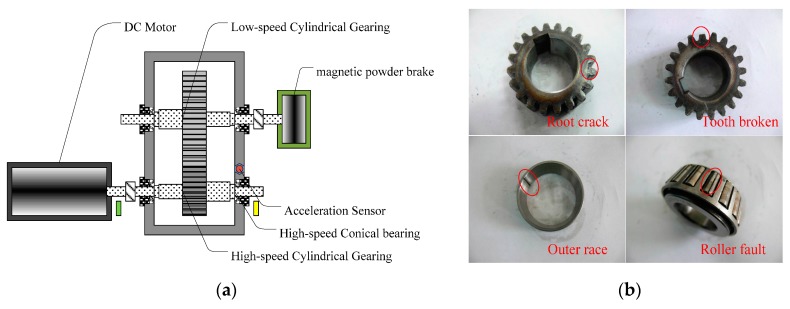
The single-stage cylindrical straight gearbox test rig: (**a**) Schematic diagram of gearbox test rig; (**b**) The damaged components.

The second dataset is from our gearbox test rig, which includes a single-stage cylindrical straight gearbox, a DC motor for driving gearbox, a magnetic powder brake for loading and data acquisition system. Four types of faults on gear and bearing are created inside the gearbox, i.e., the root crack, tooth broken, outer race fault and roller fault, respectively. Since the high-speed stage are more significant for a gearbox in terms of lifetime, all the faults are introduced to the high-speed gear and bearing. The vibration signal of gearbox casing is measured by an accelerometer with a sampling frequency 20 kHz. The speed of motor is 1500 rpm and the load is 11 N·m in the experiments. Detailed schematic diagram of test rig and damaged components are given in Figure 13.

**Figure 14:**
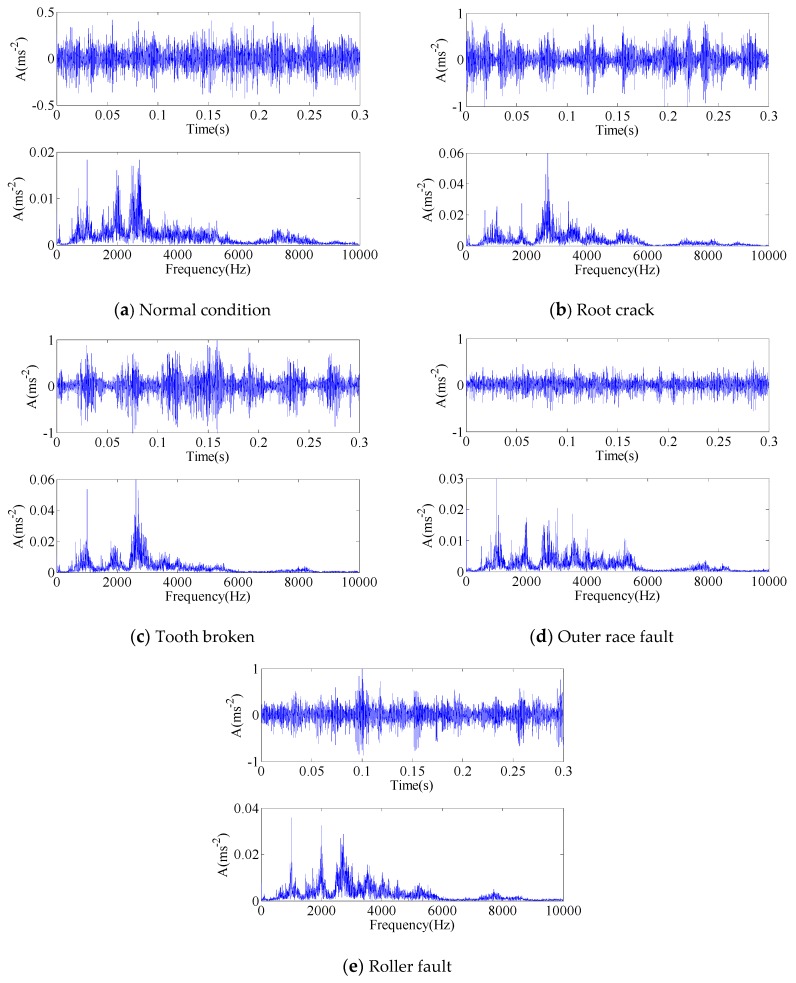
The time waveforms and frequency spectra of vibration signals: (**a**) Normal condition; (**b**) Root crack; (**c**) Tooth broken; (**d**) Outer race fault; (**e**) Roller fault.

Like the sample preparation in case 1, there are 50 samples in each data subset, from which 20 samples are split for training and the other 30 samples are tested. Each sample is a section of raw signal containing 6000 points. The time waveforms and frequency spectra of the signal samples in the five conditions are presented in Figure 14.

#### 5.2.2. Diagnosis Procedure and Results

The above-mentioned procedure is conducted on the gearbox fault datasets again. Due to the space limitations, we directly show the diagnosis results after dimensionality reduction. From Table 3, it can be find that the first three principal components can reach a relatively satisfactory contribution rate over 90% in both two methods.

**Figure 15:**
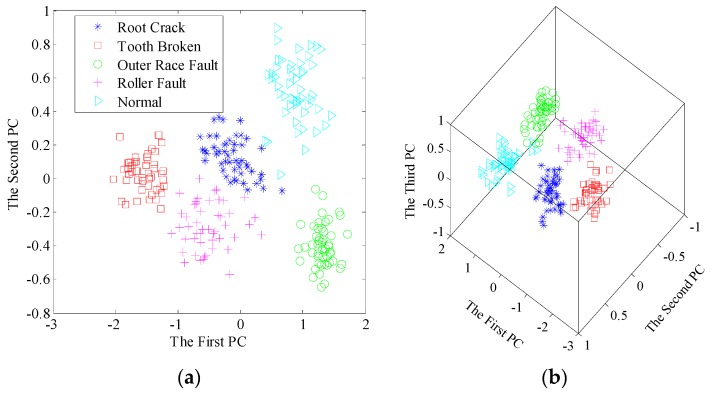
Scatter plots of the principal components after dimensionality reduction using dictionary learning and SVD: (**a**) The first two principal components; (**b**) The first three principal components.

**Figure 16:**
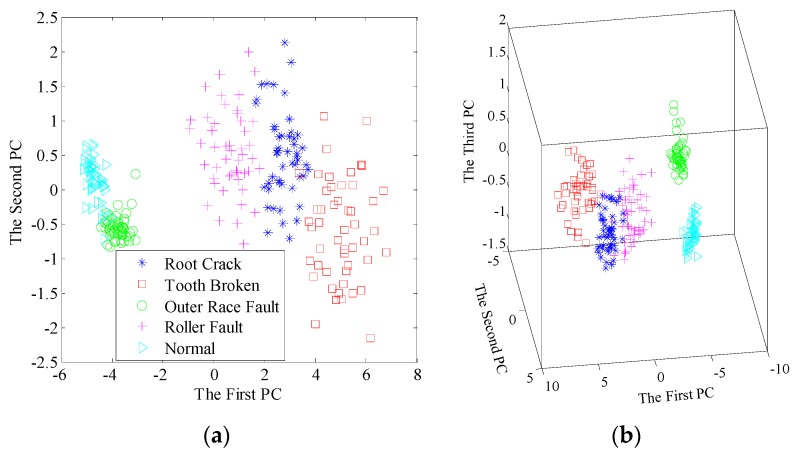
Scatter plots of the principal components after dimensionality reduction using EMD and SVD: (**a**) The first two principal components; (**b**) The first three principal components.

As shown in Figure 15, by using dictionary learning pre-processor, almost no overlapping area can be observed for the first two principal components. And furthermore, all the samples are distinctly identified for the first three principal components. For comparison, the results by EMD pre-processor are presented in Figure 16. One can see the distribution areas are very close for the three machinery conditions, namely root crack, tooth broken and roller fault, both in 2D and 3D space, which may lead to misclassification.

**Figure 17:**
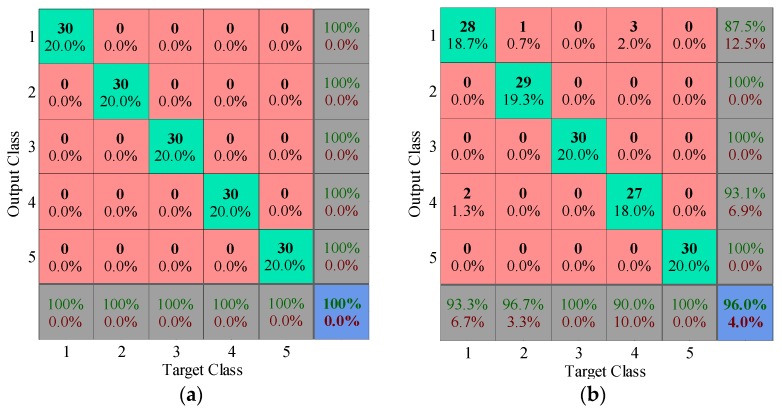
Multi-class confusion matrices of testing samples: (**a**) Dictionary learning pre-processor; (**b**) EMD pre-processor.

The diagnosis results adopting first three principal components are illustrated in Figure 17 (root crack, tooth broken, outer race fault, roller fault and normal correspond to labels 1, 2, 3, 4 and 5 respectively). Similar to scatter plots of principal components, the diagnosis accuracy using dictionary learning is 100%, while that of EMD is 96%, demonstrating the superiority of proposed method again.

## 6. Conclusions

In this research, a novel intelligent diagnosis method using dictionary learning and SVD is proposed for rotating machinery. The main idea of this framework is to extract the initial feature matrix from the raw signals by a dictionary learning scheme. Actually, the atoms learned in dictionary matrix are capable of capturing the underlying structures of analyzed signals, and thus preserve abundant identifying information. The dictionary learning scheme can extract the expected feature matrix efficiently, while avoiding the selection of lag time and embedding dimension. Afterwards, the singular value sequence is computed to denote the natural characteristic of the matrix. As the dimensionality of the sequence is high, the PCA is applied to relieve this dimensionality and generate the more significant PCs. Finally, the first several PCs are used as fault feature vectors for KNN classifier to diagnose the faults automatically. The proposed method is especially suited for classifying and recognizing the machinery conditions, which is verified by two datasets from bearing and gearbox, respectively. The comparisons with the existing EMD-based method demonstrate the superiority of this new approach.

## Figures and Tables

**Table 1:** Parameters of K-SVD dictionary learning.

Initial Dictionary	Overlap Size	Sparse Coding	Noise Level	Update Iterations
DCT	Maximum overlap ratio	OMP	Wavelet coefficient estimator	20

**Table 2:** Contribution rate of first few principal components in case 1.

Pre-Processor	First PC	First Two PCs	First Three PCs
Dictionary learning	0.881	0.955	0.976
EMD	0.952	0.988	0.994

**Table 3:** Contribution rate of first few principal components in case 2.

Pre-Processor	First PC	First Two PCs	First Three PCs
Dictionary learning	0.747	0.843	0.907
EMD	0.935	0.973	0.989

## References

[1] Guo L., Li N., Jia F., Lei Y., Lin J. (2017). A recurrent neural network based health indicator for remaining useful life prediction of bearings. Neurocomputing.

[2] Lei Y., Li N., Lin J., He Z. (2013). Two new features for condition monitoring and fault diagnosis of planetary gearboxes. J. Vib. Control.

[3] Nembhard A.D., Sinha J.K., Yunusa-Kaltungo A. (2015). Development of a generic rotating machinery fault diagnosis approach insensitive to machine speed and support type. J. Sound Vib..

[4] Yunusa-Kaltungo A., Sinha J.K. (2016). Sensitivity analysis of higher order coherent spectra in machine faults diagnosis. Struct. Health Monit..

[5] Gao Z., Cecati C., Ding S.X. (2015). A survey of fault diagnosis and fault-tolerant techniques—Part I: Fault diagnosis with model-based and signal-based approaches. IEEE Trans. Ind. Electron..

[6] Gao Z., Cecati C., Ding S.X. (2015). A Survey of Fault Diagnosis and Fault-Tolerant Techniques—Part II: Fault Diagnosis With Knowledge-Based and Hybrid/Active Approaches. IEEE Trans. Ind. Electron..

[7] Liu W., Han J., Lu X. (2013). A new gear fault feature extraction method based on hybrid time–frequency analysis. Neural Comput. Appl..

[8] Wang D., Shen C., Tse P.W. (2013). A novel adaptive wavelet stripping algorithm for extracting the transients caused by bearing localized faults. J. Sound Vib..

[9] An X., Jiang D., Li S., Zhao M. (2011). Application of the ensemble empirical mode decomposition and Hilbert transform to pedestal looseness study of direct-drive wind turbine. Energy.

[10] Chen X., Feng F., Zhang B. (2016). Weak fault feature extraction of rolling bearings based on an improved kurtogram. Sensors.

[11] Lei Y., He Z., Zi Y. (2011). EEMD method and WNN for fault diagnosis of locomotive roller bearings. Expert Syst. Appl..

[12] Lei Y., Jia F., Lin J., Xing S., Ding S.X. (2016). An intelligent fault diagnosis method using unsupervised feature learning towards mechanical big data. IEEE Trans. Ind. Electron..

[13] Li K., Zhang Q., Wang K., Chen P., Wang H. (2016). Intelligent condition diagnosis method based on adaptive statistic test filter and diagnostic bayesian network. Sensors.

[14] Li Y., Xu M., Wei Y., Huang W. (2016). A new rolling bearing fault diagnosis method based on multiscale permutation entropy and improved support vector machine based binary tree. Measurement.

[15] An X., Tang Y. (2016). Application of variational mode decomposition energy distribution to bearing fault diagnosis in a wind turbine. Trans. Inst. Meas. Control.

[16] Han T., Jiang D. (2016). Rolling bearing fault diagnostic method based on VMD-AR model and random forest classifier. Shock Vib..

[17] Elad M., Aharon M. (2006). Image denoising via sparse and redundant representations over learned dictionaries. IEEE Trans. Image Process..

[18] Bin Y., Shutao L. (2010). Multifocus image fusion and restoration with sparse representation. IEEE Trans. Instrum. Meas..

[19] Yang S., Wang M., Chen Y., Sun Y. (2012). Single-image super-resolution reconstruction via learned geometric dictionaries and clustered sparse coding. IEEE Trans. Image Process..

[20] Chen X., Du Z., Li J., Li X., Zhang H. (2014). Compressed sensing based on dictionary learning for extracting impulse components. Signal Process..

[21] Tang H., Chen J., Dong G. (2014). Sparse representation based latent components analysis for machinery weak fault detection. Mech. Syst. Signal Process..

[22] Liu H., Liu C., Huang Y. (2011). Adaptive feature extraction using sparse coding for machinery fault diagnosis. Mech. Syst. Signal Process..

[23] Zhou H., Chen J., Dong G., Wang R. (2016). Detection and diagnosis of bearing faults using shift-invariant dictionary learning and hidden Markov model. Mech. Syst. Signal Process..

[24] Guo L., Gao H., Li J., Huang H., Zhang X. (2015). Machinery vibration signal denoising based on learned dictionary and sparse representation. J. Phys. Conf. Ser..

[25] Donoho D.L., Elad M., Temlyakov V.N. (2006). Stable recovery of sparse overcomplete representations in the presence of noise. IEEE Trans. Inf. Theory.

[26] Bruckstein A.M., Donoho D.L., Elad M. (2009). From sparse solutions of systems of equations to sparse modeling of signals and images. SIAM Rev..

[27] Aharon M., Elad M., Bruckstein A. (2006). K-SVD: An algorithm for designing overcomplete dictionaries for sparse representation. IEEE Trans. Signal Process..

[28] Sulam J., Ophir B., Zibulevsky M., Elad M. (2016). Trainlets: Dictionary learning in high dimensions. IEEE Trans. Signal Process..

[29] Kilundu B., Chiementin X., Dehombreux P. (2011). Singular spectrum analysis for bearing defect detection. J. Vib. Acoust..

[30] Muruganatham B., Sanjith M.A., Krishnakumar B., Satya Murty S.A.V. (2013). Roller element bearing fault diagnosis using singular spectrum analysis. Mech. Syst. Signal Process..

[31] Han T., Jiang D., Wang N. (2016). The fault feature extraction of rolling bearing based on EMD and difference spectrum of singular value. Shock Vib..

[32] Cheng J., Yu D., Tang J. (2009). Application of SVM and SVD technique based on EMD to the fault diagnosis of the rotating machinery. Shock Vib..

[33] Luo S., Cheng J., Ao H. (2015). Application of LCD-SVD technique and CRO-SVM method to fault diagnosis for roller bearing. Shock Vib..

[34] Tian Y., Ma J., Lu C., Wang Z. (2015). Rolling bearing fault diagnosis under variable conditions using LMD-SVD and extreme learning machine. Mech. Mach. Theory.

[35] Huang N., Chen H., Cai G., Fang L., Wang Y. (2016). Mechanical fault diagnosis of high voltage circuit breakers based on variational mode decomposition and multi-layer classifier. Sensors.

[36] Yunusa-Kaltungo A., Sinha J.K., Nembhard A.D. (2015). A novel fault diagnosis technique for enhancing maintenance and reliability of rotating machines. Struct. Health Monit..

[37] Wang D., Tsui K.-L., Tse P.W., Zuo M.J. (2015). Principal components of superhigh-dimensional statistical features and support vector machine for improving identification accuracies of different gear crack levels under different working conditions. Shock Vib..

[38] Van Der Maaten L., Postma E., van den Herik J. (2009). Dimensionality reduction: A comparative review. J. Mach. Learn. Res..

